# Donor-Site Morbidity Analysis of Thenar and Hypothenar Flap

**DOI:** 10.1055/a-2168-4771

**Published:** 2024-02-07

**Authors:** Dong Chul Lee, Ho Hyung Lee, Sung Hoon Koh, Jin Soo Kim, Si Young Roh, Kyung Jin Lee

**Affiliations:** 1 Department of Plastic and Reconstructive Surgery, Gwangmyeong Sungae General Hospital, Gwangmyeong, Republic of Korea

**Keywords:** free thenar flap, RASP free flap, hypothenar free flap, donor-site morbidity

## Abstract

**Background** For the small glabrous skin defect, Thenar and Hypothenar skin are useful donors and they have been used as a free flap. Because of similar skin characteristics, both flaps have same indications. We will conduct comparative study for the donor morbidity of the Free thenar flap and Hypothenar free flap.

**Methods** From January 2011 to December 2021, demographic data, characteristics of each flap, and complications using retrospective chart review were obtained. Donor outcomes of the patient, who had been followed up for more than 6 months, were measured using photographic analysis and physical examination. General pain was assessed by Numeric Rating Scale (NRS) score, neuropathic pain was assessed by Douleur Neuropathique 4 Questions (DN4) score, scar appearance was assessed by modified Vancouver Scar Scale (mVSS), and patient satisfaction was assessed on a 3-point scale. Statistical analysis was performed on the outcomes.

**Results** Out of the 39 survey respondents, 17 patients received Free thenar flaps, and 22 patients received Hypothenar free flaps. Thenar group had higher NRS, DN4, and mVSS ( *p* < 0.05). The average scores for the Thenar and Hypothenar groups were 1.35 and 0.27 for NRS, 2.41 and 0.55 for DN4, and 3.12 and 1.59 for mVSS, respectively. Despite the Hypothenar group showing greater satisfaction on the 3-point scale (1.82) compared with the Thenar group (1.47), the difference was not significant ( *p* = 0.085). Linear regression analysis indicated that flap width did not have a notable impact on the outcome measures, and multiple linear regression analysis revealed no significant interaction between flap width and each of the outcome measures.

**Conclusion** Despite the limited number of participants, higher donor morbidity in general pain, neuropathic pain, and scar formation was noted in the Thenar free flap compared with the Hypothenar free flap. However, no difference in overall patient satisfaction was found between the two groups.

## Introduction

Using glabrous skin in coverage for hand defect offers the advantage of a thicker epidermis, which enables the skin to withstand increased force, pressure, and shear. 1 Specifically, glabrous skin receptors facilitate tactile feedback through sensory-motor memory adaptation and input, providing functional benefits for digits. 2 Based on these properties, small free flaps such as the Free Thenar flap, Hypothenar free flap, and toe pulp free flap are used in digit reconstruction. 3 4 5 6

Among these, the Free thenar flap and Hypothenar free flap are utilized with similar donor sites and indications. The Free thenar flap can be harvested longitudinally from the thenar eminence toward the scaphoid tubercle in the palm's midline, typically using the superficial palmar branch of the radial artery as the source vessel. 7 The hypothenar free flap can be harvested from within the hypothenar eminence in various sizes and shapes based on the selection of different perforators. 4 8 9

Both flaps are harvested from the palm, allowing primary closure of the donor site and having a limited size available, which emphasizes the need to minimize donor morbidity. 10 Therefore, the aim of this study is to compare donor morbidity through an analysis of prognosis and to describe the characteristics of each flap with an evaluation of risk factors.

## Methods

This study was a retrospective chart review. The protocol was approved by our hospital's Ethics Review Board (IRB no. KIRB 2023-N-002), and informed consent for undergoing procedures was obtained from all patients.

### Inclusion Criteria

Patients who (1) underwent Free thenar flap and Hypothenar free flap, (2) had follow-up data for more than 6 months, allowing for sufficient maturation of the donor site scar, and (3) visited our hospital between January 2011 and December 2020.

### Exclusion Criteria

Patients who used (1) a skin graft without primary closure on the donor site and (2) the proximal ulnar artery perforator and harvested longitudinally from the proximal hypothenar eminence for Hypothenar free flap cases.

### Study Design

**Fig. 1:**
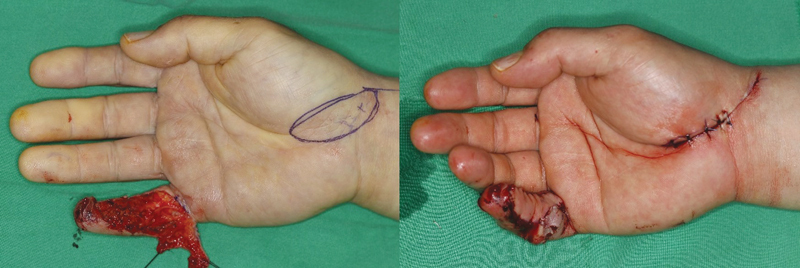
A 51-year-old man used innervated radial artery superficial palmar branch perforator free flap to coverage soft tissue defect of right little finger.

We conducted data collection for patients who met the above criteria. Each patient group was defined as follows:

The Free thenar flap group was limited to those using the radial artery superficial palmar branch (RASP) perforator and harvested longitudinally in a conventional manner at the wrist crease ( Fig. 1 ). 11

**Fig. 2:**
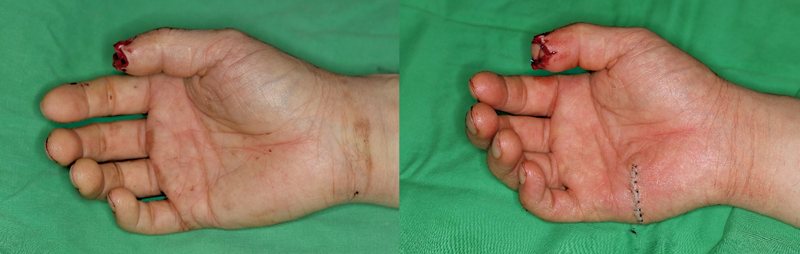
A 40-year-old man used Hypothenar perforator free flap to coverage soft tissue defect of right little finger.

The Hypothenar free flap can be harvested longitudinally from the proximal thenar eminence or transversely from the palmar crease. In this study, we included only the group that harvested the Hypothenar free flap parallel to the palmar crease using the fourth common digital artery perforator free flap method described by Safa et al ( Fig. 2 ). 8

Only cases where the donor site was primarily closed were included, and those who received skin grafts or other treatments due to dehiscence were excluded because it can alter the appearance of the scar.

Patients' demographic data, flap width, and flap length were collected through chart review. Donor-site morbidity included pain, neurogenic symptoms, scar formation, and patient satisfaction, and was measured comprehensively using a questionnaire and physical exam for patients who visited the outpatient clinic. To perform a quantitative analysis of the outcome, we established the following criteria for each respective aspect.

1. General pain was measured using the Numeric Rating Scale (NRS) score, ranging from 0 to 10. This scale, commonly used for pain screening, has modest accuracy in identifying patients with clinically significant pain in primary care settings. The survey was conducted on outpatient visitors. 12
2. Neuropathic pain was assessed using the Douleur Neuropathique 4 Questions (DN4) score, which ranges from 0 to 10. It demonstrates high sensitivity and specificity, enabling the differentiation and determination of the severity of chronic neuropathic pain from non-neuropathic pain. Data were collected through surveys and physical examinations conducted on outpatient clinic visitors. 13 14
3. Scar appearance was assessed using the modified Vancouver Scar Scale (mVSS), which is a method that evaluates vascularity, height/thickness, pliability, and pigmentation through a total scoring system of 16 points. Though unsuitable for large, heterogeneous scars, the mVSS was adopted due to its advantage of minimizing the impact of patients' perception. Assessments were conducted using photography and direct physical examination of outpatient visitors. 15 16 17
4. Patient satisfaction was assessed using a 3-point scale survey, concentrating on the donor site with scores ranging from 0 to 2. The study utilized patient satisfaction as an independent outcome to explore patient perception, excluding the influence of the reconstruction surgery prognosis.

### Statistical Analysis

Patient's demographic data, width, length of the flap were included. NRS and DN4 scores, mVSS, and 3-point scale of patients satisfaction were evaluated as outcomes.

A comparative prognostic analysis was conducted using an independent *t* -test between the two groups. Additionally, a linear regression test was performed between each outcome and flap width, and for horizontal correction, multiple linear regression was conducted for each group.

The statistical analysis was performed with IBM SPSS Statistics for Windows, version 26.0 (IBM Corp., Armonk, NY) using the regression test, *p* -value of less than 0.05 was considered statistically significant.

## Results

A total of 39 patients, out of 76, who underwent Free thenar flap or Hypothenar free flap surgery from January 2011 to December 2020 responded to a survey. Of these patients, 17 were in the Free thenar flap group and 22 were in the Hypothenar free flap group, 31 were males and 8 were females, with an average age of 45.69 years. The harvested flap width was wider in the Free thenar flap group, with a mean of 1.96 mm, compared with 1.58 mm in the Hypothenar free flap group. The mean length was also longer (4.43 mm) in the Free thenar flap group, compared to 2.93 mm in the Hypothenar free flap group. The presence of nerves in the harvested flap was observed in seven cases in the Free thenar flap group and two cases in the Hypothenar free flap group ( Table 1 ).

**Table 1:** Patient demographics

	Free thenar flap ( *n* = 17)	Hypothenar free flap ( *n* = 22)	*p* -Value a
Age (y, median)	49.17	43	0.203
Sex			
Male	14	17	0.508
Female	3	5	–
Flap width (mm)	1.96	1.58	0.003 b
Flap length (mm)	4.43	2.93	0.010 b
Innervated flaps	7	2	0.024 b

a Chi-square test and independent *t* -test.

b *p* -value <0.05 indicates statistical significance.

An independent *t* -test was performed to compare the outcomes between the Free thenar flap and Hypothenar free flap groups. The mean values (M) and standard deviation (SD) were measured and compared for each outcome between the groups. The *t* -value (T) measures how far the difference between group means is from 0. A higher *t* -value indicates a more significant difference between the means. The results demonstrated that the hypothenar group had significantly lower NRS and DN4 scores, and mVSS, which were statistically significant ( *p* < 0.05). However, there was no significant difference between the two groups regarding overall satisfaction based on a 3-point appearance assessment ( Table 2 ).

**Table 2:** A comparison analysis of outcomes between the two groups

	Type of the flap	M	SD	*T*	*p* -Value a
NRS score	Free thenar flap	1.35	1.16	3.37	0.002 b
	Hypothenar free flap	0.27	0.7		
DN4 score	Free thenar flap	2.41	1.46	4.62	<0.001 b
	Hypothenar free flap	0.55	0.91		
mVSS	Free thenar flap	2.06	0.74	3.87	<0.001 b
	Hypothenar free flap	1.18	0.66		
3-point appearance	Free thenar flap	1.47	0.72	−1.79	0.085
	Hypothenar free flap	1.82	0.4		

Abbreviations: DN4, Douleur Neuropathique 4 Questions; M, mean value; mVSS, modified Vancouver Scar Scale; NRS, Numeric Rating Scale; SD, standard deviation; T, *T* -value.

a Independent *t* -test.

b *p* -value <0.05 indicates statistical significance.

The linear regression test results aimed to measure the relationship between flap width and each outcome. The correlation between flap width and the mVSS was found to be significant ( *p* < 0.05). The β-coefficient (β), which is the standardized regression coefficient, was 0.338, a positive value, indicating that as the flap width increased, there was an increase in scar morbidity. However, the NRS and DN4 scores, and patients' satisfaction survey did not show statistical significance in relation to flap width ( Table 3 ).

**Table 3:** Regression analysis between flap width and outcomes

	B	β	*T*	*p* -Value a
NRS score	0.109	0.062	0.379	0.707
DN4 score	0.661	0.270	1.705	0.097
mVSS	0.703	0.338	2.182	0.036 b
3-point appearance	−0.137	−0.144	−0.888	0.381

Abbreviations: B, unstandardized regression coefficient; DN4, Douleur Neuropathique 4 Questions; mVSS, modified Vancouver Scar Scale; NRS, Numeric Rating Scale; T, *T* -value; β, standardized regression coefficient.

a Linear regression test.

b *p* -value <0.05 indicates statistical significance.

Multiple linear regression tests were conducted for meaningful outcomes (NRS and DN4 scores, and mVSS) to account for the influence of flap width and identify horizontal effects. The results showed that the type of the flap was more significant than flap width ( *p* < 0.05), with larger absolute values of the β-coefficients (β) for the NRS and DN4 scores, and mVSS in all the cases. In other words, the type of flap has a greater influence on the outcome. The variance inflation factor was less than 10, indicating no multicollinearity. Therefore, it can be concluded that regardless of flap width, the thenar free flap showed worse outcomes in terms of the NRS score, DN4 score, and mVSS ( Table 4 ).

**Table 4:** Multiple linear regression test of variables

Model variables		B	β	*T*	*p* -Value a	Tolerance	VIF	*R* ^2^
1	NRS score							
	Flap type	−1.290	−0.606	−3.849	<0.001 b	0.790	1.265	0.294
	Flap width	−0.377	−0.215	−1.368	0.180			
2	DN4 score							
	Flap type	−−1.896	−0.637	−4.362	<0.001 b	0.790	1.265	0.393
	Flap width	−0.054	−0.022	−0.150	0.882			
3	mVSS							
	Flap type	−1.437	−0.568	−3.810	0.001 b	0.790	1.265	0.369
	Flap width	0.162	0.078	0.521	0.521			

Abbreviations: B, unstandardized regression coefficient; DN4, Douleur Neuropathique 4 Questions; mVSS, modified Vancouver Scar Scale; NRS, Numeric Rating Scale; *R* ^2^ , coefficient of determination; T, *T* -value; VIF, variance inflation factor; β, standardized regression coefficient.

a Multiple linear regression test.

b *p* -value < 0.05 indicates statistical significance.

## Discussion

In volar pulp and fingertip reconstruction, covering the defect with pliability and adequate fat tissue padding is important, but functional capacity is also crucial, and a glabrous skin flap provides several advantages. Glabrous skin has a durable, thicker epidermis containing a well-defined stratum lucidum, thick stratum spinosum, and corneum. Furthermore, it has developed sensory receptors that enable neurosensory feedback, which is essential for the recovery of tactile response and grasp during rehabilitation. 2 Additionally, when used in dermal grafting, it is known to have minimal donor morbidity compared with hair-bearing skin. 1 However, the donor sites of the glabrous skin are limited to the palm, plantar, and finger and toe pulp, which restricts the size available for harvest. Through this study on donor site morbidity, we aim to compare the characteristics of two glabrous skin flaps originating from the palm and elucidate their applications in reconstruction. 10

### Free Thenar Flap

The thenar area has been suggested as a potential donor site for free vascularized tissue transfer since its description by Tsai et al and Kamei et al. 3 18 It has the advantage of having consistent perforators and is useful for digit revascularization and soft tissue coverage. The RASP perforator free flap, as proposed by Yang et al, is an advanced concept that offers the added advantage of consistently harvesting the palmar cutaneous branch of the median nerve. Although differences exist between the two methods, in this study, we included those parts where the dimensions of the donor site were similar. For our research, we adopted the RASP free flap design, which involves harvesting from the interthenar region longitudinally toward the scaphoid on the radial side of the ring finger ( Fig. 1 ). 11 19

### Hypothenar Free Flap

Since Omokawa et al first proposed it as a vascularized free flap, the Hypothenar free flap has been used as a functionally and aesthetically superior reconstruction method. 20 Kim et al reported that there may be diverse perforators on the hypothenar eminence, allowing for longitudinal harvesting from the proximal thenar eminence and transverse harvesting from the distal portion. 5 According to research by Safa et al, the fourth common digital artery perforator-based (FCDAP) flap has relatively constant vascular anatomy. In this study, the FCDAP flap was adopted, harvesting a versatile flap parallel to the palmar crease of the distal hypothenar area ( Fig. 2 ). 8 9

The analysis demonstrated that the Hypothenar free flap had lower rates of somatic pain, neurogenic symptoms, and scar formation compared with the Free thenar flap, after adjusting for horizontal correction with flap width using multiple linear regression. In other words, for small defects with similar clinical situations, the Hypothenar perforator free flap may be the flap of choice. Additionally, when adopting the thenar free flap for benefits such as sensory recovery, efforts should be made to reduce donor morbidity through precise design to minimize flap size, a thorough understanding of anatomy, and careful placement of surgical retractors with meticulous dissection to avoid injury to nearby structures, including the superficial branch of the radial artery. 21 22

The potential reasons for this result include the thenar area being more noticeable and tactilely perceived in daily life, making any discomfort or inconvenience more apparent than in the hypothenar area. Additionally, thumb movement in the Free thenar flap can cause pain due to the hyperabduction position of the first carpometacarpal joint. Conversely, the Hypothenar free flap had temporary postoperative little finger extension limitation that resolved within 2 to 3 weeks, with no complaints about range of motion limitation after 6 months.

**Fig. 3:**
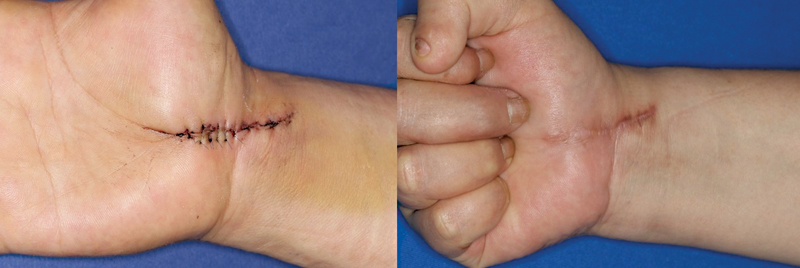
Follow-up photograph of the 42-year-old man who underwent Free thenar flap, postoperative 7 days and 7 months later. The patient had no neuropathic pain but showed hypertrophic scarring (NRS score: 1, DN4 score: 0, mVSS: 2, 3-point scale: 2). DN4, Douleur Neuropathique 4 Questions; mVSS, modified Vancouver Scar Scale; NRS, Numeric Rating Scale.

**Fig. 4:**
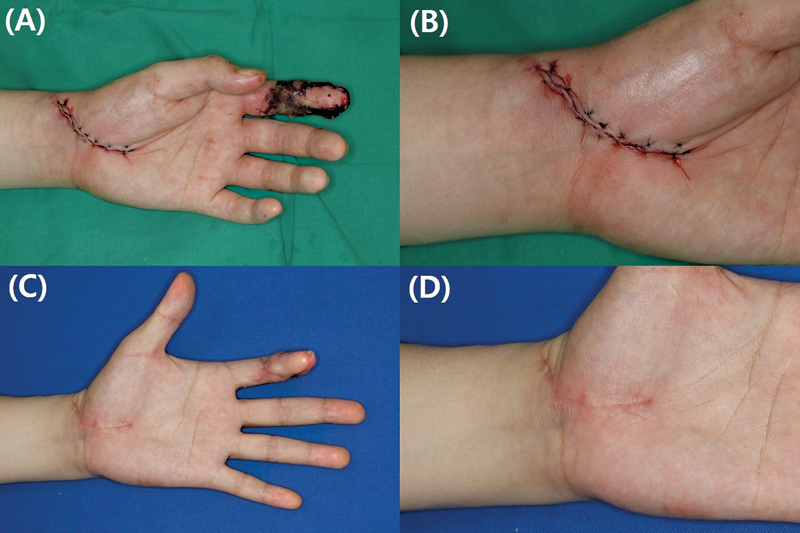
Follow-up photographs of a 35-year-old man who underwent a Free thenar flap: ( **A, B** ) immediate postoperative images and ( **C, D** ) images taken after 1 year. Scar formation was relatively more prominent in the nonglabrous skin area located proximal to the wrist crease (NRS score: 1, DN4 score: 3, mVSS: 3, 3-point scale: 2). DN4, Douleur Neuropathique 4 Questions; mVSS, modified Vancouver Scar Scale; NRS, Numeric Rating Scale.

**Fig. 5:**
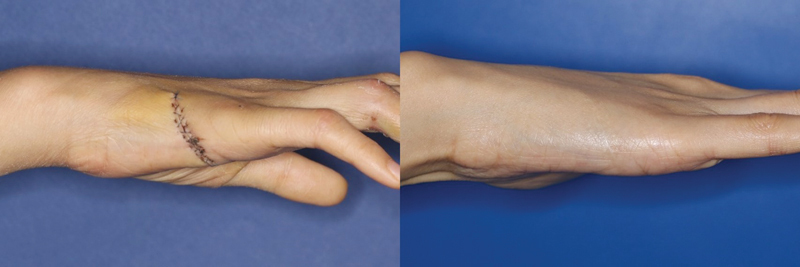
Follow-up photograph of the 30-year-old woman who underwent Hypothenar perforator free flap, postoperative 7 days and 6 months later. The patient had minimal neuropathic symptoms and almost invisible scarring (NRS score: 0, DN4 score: 1, mVSS: 1, 3-point scale: 2). DN4, Douleur Neuropathique 4 Questions; mVSS, modified Vancouver Scar Scale; NRS, Numeric Rating Scale.

**Fig. 6:**
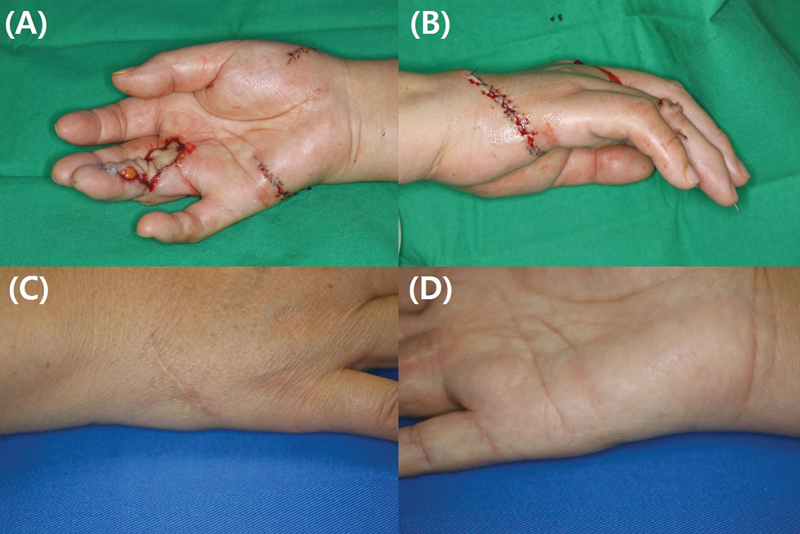
Follow-up photograph of the 51-year-old woman who underwent Hypothenar perforator free flap: ( **A, B** ) immediate postoperative images and ( **C, D** ) images taken after 1 year. Scar formation from the donor site is more likely to occur on the nonglabrous skin of the hand dorsum (NRS score: 0, DN4 score: 2, mVSS: 2, 3-point scale: 2). DN4, Douleur Neuropathique 4 Questions; mVSS, modified Vancouver Scar Scale; NRS, Numeric Rating Scale.

The extent of glabrous skin included in the results may have influenced the outcome. The Free thenar flap is harvested from the interthenar space to the wrist and distal forearm to search for the source vessel of the perforator, causing the donor scar to be directly affected by wrist movement. Furthermore, more scar formation was observed on the distal forearm compared with the glabrous skin of the palm ( Figs. 3 and 4 ). In contrast, the Hypothenar free flap exhibited less scar formation relative to the Free thenar flap. Within the same donor, scar formation tended to increase as the donor site extended toward the nonglabrous skin of the hand dorsum ( Figs. 5 and 6 ).

In addition, a comparison study of the donor harvest size and transfer rate between the two groups was conducted, which revealed that the hypothenar area had a width of 3 cm for primary closure, and if the width was wider, skin grafting was required due to flap limitation. In contrast, the thenar area had a wider flap elevation range due to the possibility of skin approach in thumb opposition position after flap harvest. If the clinical situation requires more extensive coverage and sensory recovery, the Free thenar flap can be considered.

The limitations of this study are as follows. First, in the thenar area, a single perforator from the radial artery's superficial palmar branch is typically used, but sometimes it may be absent, requiring a search for a direct perforator from the radial artery. In the hypothenar area, perforators vary by location, and different authors describe them differently. This study included cases using the fourth common digital artery perforator in the midportion as a reference. If different perforators are used and the flap's axis and design change, results may differ. 8

Second, hand size was not standardized. Women's skin is generally more pliable, and men's hands are usually larger, allowing for larger, thicker flaps. Flap width was used for correction at the primary closure site in this study, but bias may exist, and more research with larger populations is needed.

Third, recipient conditions were not identical. Reconstruction prognosis and satisfaction showed no significant differences between groups, possibly influencing the overall reconstruction outcome. This implies that factors such as the state of the defect to be reconstructed, the practical usage of the patient's digits, and their occupation have an impact. A better study could involve a prospective comparative study of the same recipient and reconstruction prognosis.

The Hypothenar perforator free flap tends to have lower donor morbidity compared with the Free thenar flap when they are of the same size, but the latter allows for larger harvests and nerve inclusion ( Table 5 ).

**Table 5:** The summary of the advantages and disadvantages of two groups

	Free thenar flap	Hypothenar free flap
Advantages	Sensate free flap Wider harvest	Low donor morbidity
Disadvantages	Higher donor morbidity	Small flap size Perforator variation
